# Design and Validation of a Multimodal Diffuse Reflectance and Spatially Offset Raman Spectroscopy System for In Vivo Applications

**DOI:** 10.1002/jbio.202400333

**Published:** 2024-12-25

**Authors:** April Mordi, Varsha Karunakaran, Umme Marium Mim, Eric Marple, Narasimhan Rajaram

**Affiliations:** ^1^ Department of Biomedical Engineering University of Arkansas Fayetteville Arkansas USA; ^2^ EmVision LLC Loxahatchee Florida USA

**Keywords:** diffuse reflectance spectroscopy, multimodal spectroscopy, Raman spectroscopy, spatially offset Raman spectroscopy

## Abstract

We report on the development of a multimodal spectroscopy system, combining diffuse reflectance spectroscopy (DRS) and spatially offset Raman spectroscopy (SORS). A fiber optic probe was designed with spatially offset source–detector fibers to collect subsurface measurements for each modality, as well as ball lens‐coupled fibers for superficial measurements. The system acquires DRS, zero‐offset Raman spectroscopy (RS) and SORS with good signal‐to‐noise ratio. Measurements on chicken breast tissue demonstrate that both DRS and RS can acquire spectra from similar depths within tissue. Measurements acquired from the skin of a human volunteer demonstrate distinct Raman peaks at 937 and 1755 cm^−1^ that were unique to the zero‐offset ball lens configuration and 718 and 1089 cm^−1^ for the spatially offset setting. We also identified Raman peaks corresponding to melanin that were prominent in the superficial measurements obtained with the ball lens‐coupled fibers but not in the spatially offset fibers.

## Graphical abstract

This work presents the combination of diffuse reflectance spectroscopy and spatially offset Raman spectroscopy into a multimodal system with a fiber optic handheld probe and optical switch. The probe was designed with a spatially offset source–detector configuration to collect subsurface measurements for each modality, as well as ball lens‐coupled fibers for surface measurements. The results of validation studies using the system with measurements on a chicken breast tissue phantom consisting of fat and muscle tissue, as well as measurements acquired from the skin of a human volunteer, are shown.

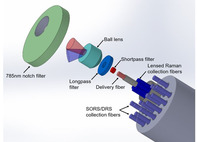

## 1 Introduction

Most cancer patients are treated with surgery in combination with some other curative therapy such as radiation, chemotherapy, or immunotherapy. Monitoring response to therapy is critical to improve response rates; however, detecting response to these treatments during or shortly after therapy is limited by current techniques. Methods to detect response to therapy are either invasive, such as oxygen‐sensing pO_2_ microelectrodes [1, 2, 3], are used several weeks to months after the course of treatment, such as positron emission tomography (PET) [4, 5], or expose the patient to more ionizing radiation, such as computed tomography (CT) [6]. Additionally, biopsies are not performed as part of current clinical practice during therapy. Therefore, there are no methods to detect response during the treatment regimen. As a result, patients whose tumors are not responding successfully to therapy may end up continuing with an unfavorable treatment plan, rather than being transferred to a modified approach that may lead to better outcomes.

Diffuse reflectance spectroscopy (DRS) offers the ability to noninvasively interrogate accessible tumors during therapy. DRS is sensitive to elastically scattered light and absorption within tissue. Prominent tissue scatterers—cells, cell nuclei, cell mitochondria, and collagen, and absorbers—oxygenated and deoxygenated hemoglobin—undergo significant changes during tumor progression [7, 8] and crucially in response to treatment [9, 10]. Quantifying the light interaction with these biological constituents provides an opportunity to noninvasively determine the treatment response. Tabassum et al. [11] showed that the magnitude and slope of the wavelength‐dependent scattering coefficient are strongly correlated with markers of apoptosis in animal models of prostate and breast cancer treated with chemotherapy. Measurements of vascular oxygenation, which is calculated based on the ratio of oxygenated to total hemoglobin, have been shown to be sensitive to radiation‐induced changes within the tumor microenvironment [12, 13, 14, 15, 16]. Troncoso et al. [17] found significant differences in reoxygenation trends in tumors treated with different immune checkpoint inhibitors.

However, the measurement of tumor oxygenation exclusively may not be a sufficient indicator of treatment response as radiation‐resistant tumors have also shown reoxygenation after radiation [13, 14, 15, 18]. Therefore, there is a need for techniques that are sensitive to treatment‐induced tumor microenvironmental changes that can be investigated alongside tumor vascular oxygenation to provide a more complete picture of treatment‐induced changes that drive response and resistance.

Raman spectroscopy (RS) has been used in several applications geared toward clinical translation. This technique is based on the Raman effect, in which light is inelastically scattered upon interaction with biological specimens. The Raman shift is unique to every molecule, making it highly specific to biomolecular changes in the tumor microenvironment. With conventional fiber‐based RS, a ball lens is placed in front of the optical fibers to focus the illumination, and inelastically scattered light from the specimen is acquired from a very shallow depth [19]. Harder et al. [20] used Raman spectral analysis in combination with principal component analysis to detect radiation‐induced changes in human non‐small‐cell lung cancer xenografts, in which they found Raman signatures that could be linked to nucleic acids, lipids, proteins and carbohydrates. We have shown that there are statistically significant differences in lipid and collagen‐like features between radiation‐resistant and sensitive tumors [21]. Furthermore, we have shown that RS coupled with machine learning approaches is able to distinguish between tumors treated with different immune checkpoint inhibitors [22]. However, these measurements have typically been performed after completion of therapy and tumor excision. Spatially offset Raman spectroscopy (SORS) separates the illumination and collection zones and is thus able to probe deeper within tissue [23, 24, 25]. While it has not been used to detect response to therapy, SORS has been used in several other applications including cancer diagnosis [26, 27, 28] and surgical margin evaluation [29, 30, 31]. Stone et al. [26] used SORS to detect biochemical information from calcifications, which indicate the possible presence of cancerous breast lesions depending on their composition, in order to distinguish between benign and malignant calcifications at clinically relevant depths. The same group also utilized transmission RS, a SORS‐related technique, to identify and characterize such calcifications at greater depths [32, 33, 34].

The overall goal of this study was to develop and validate a multimodal spectroscopy system that provides complementary biological information from tissue. We describe a system that combines DRS and RS to enable noninvasive sensing of changes in vascular oxygenation as well as associated biomolecular/microenvironmental changes from similar depths in tissue. We designed a fiber optic probe with a spatially offset source and detector fibers at different distances for both modalities to enable sub‐diffuse acquisition at similar depths. Our results show that we can acquire diffuse reflectance and Raman spectra beyond the sample surface. The main innovation is in our probe design, which contains spatially offset configurations for both RS and DRS, as well as lensed collection fibers which can be used to acquire spectra from shallow depths. We describe the process of assembling the system, acquiring spectral measurements and analyzing the collected data, as well as validation studies conducted using the system.

## 2 Materials and Methods

### 2.1 Instrumentation

#### 2.1.1 System Setup

**FIGURE 1:**
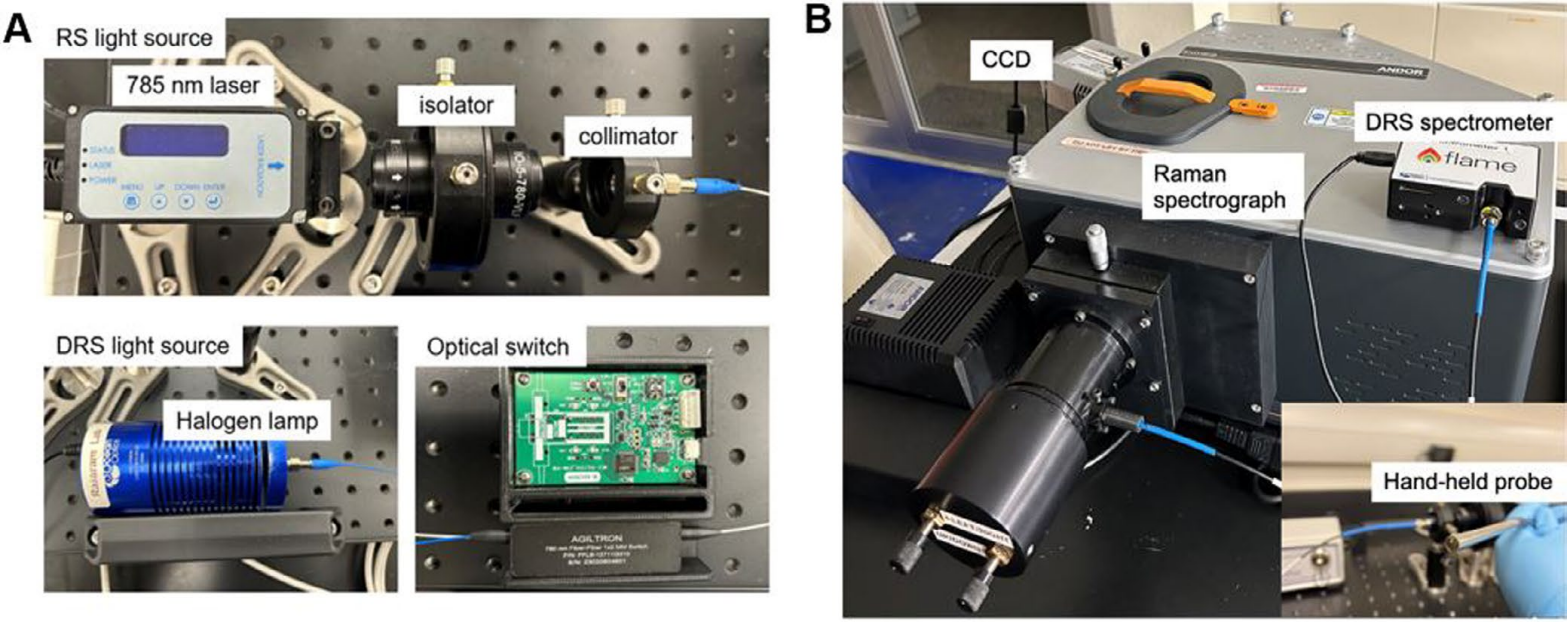
Components used in the multimodal spectroscopy system for diffuse reflectance and Raman spectroscopy. (A) 785 nm laser and halogen lamp used as RS and DRS light sources, respectively, isolator and collimator used in the light path, and optical switch used in the multimodal spectroscopy system. (B) Raman spectrograph and CCD, USB spectrometer for DRS, and hand‐held fiber optic probe used in the multimodal spectroscopy system.

The DRS and RS components of the multimodal system are shown in Figure 1. The multimodal system consists of a 785 nm laser module (LM‐785‐PLR‐225, Coherent Inc., Saxonburg, PA) for Raman excitation and a tungsten halogen lamp (HL‐2000, Ocean Optics, Dunedin, FL) for white light illumination. The output power of the laser can be controlled by adjusting the automatic current control (ACC current) either via the laser module or code written in MATLAB. A free‐space isolator (IO‐5‐780‐VLP, Thorlabs Inc., Newton, NJ) was placed at the exit of the laser to facilitate the propagation of light in one direction and prevent back reflection of the laser beam. The laser beam was also collimated by an FC/PC fiber collimator (F240FC‐780, Thorlabs Inc., Newton, NJ) to narrow the beam of light entering the fibers of the probe. Overall, this helps to reduce the loss of power as the light source is a free space laser and not directly fiber‐coupled to the rest of the system. A portable USB spectrometer (Flame, Ocean Optics, Dunedin, FL) was used for acquisition of diffusely reflected light in the wavelength range of 475–600 nm as described previously [16, 35, 36]. Raman spectra were acquired with a 328 mm focal length, motorized Czerny‐Turner spectrograph with an f/4.1 aperture (Kymera 328i, Andor Technology, Belfast, UK) coupled to a charge coupled device (CCD) camera (1024 × 256 pixel array, iDus 420, Andor Technology, Belfast, UK). The focusing mirror tube coupled to the side entrance port of the spectrograph has *x*–*y* adjustment screws. These allow for horizontal and vertical movement of the image, respectively, and alignment of the image on the entrance slit, which is useful for intensity calibration. Prior to data acquisition, the Raman CCD is cooled to −70°C. A 2 × 1 optical switch (Fiber‐Fiber Large Core Fiber Optical Switch, Agiltron Inc., Woburn, MA) with a compatible switch driver is used to alternate between the DRS and RS light sources. The switch contains two optical fiber inputs, connected to the halogen lamp and the 785 nm laser, respectively, and a single optical fiber output which is coupled to the illumination fiber of the optical probe via a SMA‐to‐SMA mating sleeve. Each of the optical fibers of the switch has a core diameter of 300 μm and is terminated by an SMA connector. The switch is bidirectional, and the unit can be controlled via a manual push button, by standard transistor–transistor logic (TTL) signal, or through USB with graphic software to change the light path.

#### 2.1.2 Probe Design

**FIGURE 2:**
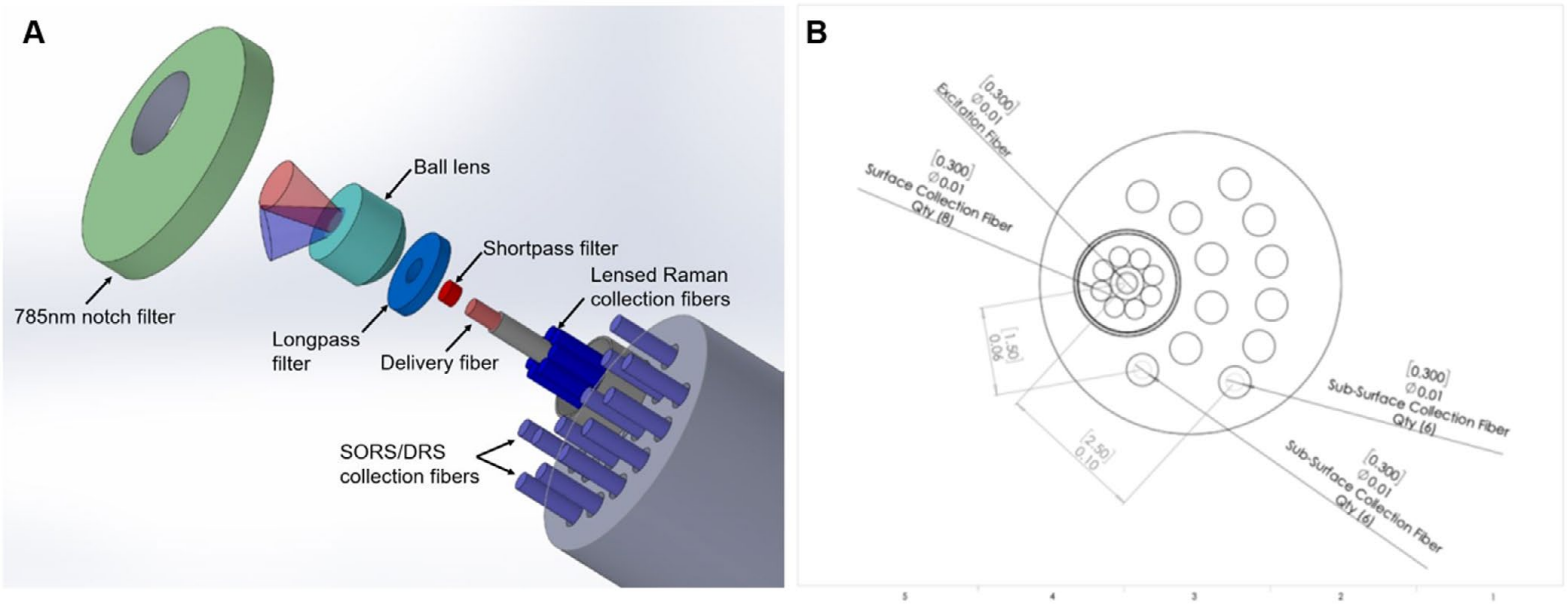
(A) Exploded view of the tissue end of the probe assembly showing lens, fiber filters, and light delivery and collection fibers for lensed Raman spectroscopy and spatially offset Raman and diffuse reflectance spectroscopy. (B) Front‐facing view of the probe displaying fiber alignment and sizes for each set of fibers used in the probe.

Figure 2A presents an exploded view of the tissue end of the fiber optic probe showing the fiber filters, two‐component ball lens, collection fibers, and excitation fiber. All optical fibers have a core diameter of 300 μm, as seen in Figure 2B, and all these fibers have a 500‐μm spot size at the distal surface of the probe. The probe consists of a four‐legged design with SMA terminations on each end which are connected to the respective excitation sources and collection spectrometers. One leg of the custom‐made fiber optic probe (EmVision LLC, Loxahatchee, FL) contains a central short pass filtered (93% transmission below 792 nm, OD 6 blocking 808–1200 nm) fiber for excitation from the Raman laser or white light source. The second leg contains six collection fibers for surface measurements with a longpass filter (93% transmission from 812 to 1200 nm, OD 6 blocking 790 nm and shorter) in front of them at the distal tip to reject the laser excitation light and only allow through the Raman scattered light. While there is a physical spatial offset of this lensed fiber, the spot position of the excitation and collection overlap at the distal face with no offset. This second leg has the fibers oriented in a line at the SMA connector. The third leg of the probe contains six collection fibers radially located at 1.5 mm from the source fiber with a notch filter (93% transmission from 400 to 742 nm and 828 to 1200 nm, OD 6 blocking at 785 nm) in front of them at the distal tip to reject the laser excitation light and only allow through the Raman scattered light that are used for subsurface Raman measurements (or diffuse reflectance measurement), and one collection fiber from the surface measurement collection portion. This third leg has the fibers oriented in a line at the SMA connector. The fourth leg of the probe consists of six collection fibers radially located at 2.5 mm from the source fiber with a notch filter (93% transmission from 400 to 742 nm and 828 to 1200 nm, OD 6 blocking at 785 nm) in front of them at the distal tip that are used for subsurface Raman measurements (or diffuse reflectance measurement), and one collection fiber from the surface measurement collection portion. This fourth leg has the fibers oriented in a line at the SMA connector. The SMA connector for each set of fibers contains the six spatially offset fibers as well as one 300‐μm core fiber from the lensed surface measuring probe. The zero offset (ball lens) collection fibers integrated within Leg 3 and Leg 4 can be used to simultaneously acquire spectra from the surface in addition to the spatially offset Raman spectra. The availability of this zero‐offset fiber has advantages when sampling tissue where the availability of information at multiple depths can provide better discriminating information. While we use the fibers in the third leg (1.5 mm source–detector offset) for RS and fibers in the fourth leg (2.5 mm source–detector offset) for DRS to obtain approximately similar sampling depths, it is important to note that each leg is capable of both DRS and RS measurements.

### 2.2 System Calibration

Prior to the collection of sample spectra, spectra for calibration and background subtraction are acquired daily. For both DRS and RS, background spectra are measured with the probe placed in a black test tube. For background measurements with RS, the laser is turned off using MATLAB code. Background spectra for DRS are acquired by placing an absorptive filter (NE260B, Thorlabs Inc., Newton, NJ) with an optical density of 6.0 in the light path. This is necessary because the halogen lamp lacks a TTL port that can be addressed via software to turn it off. We have confirmed that the OD 6.0 filter effectively eliminates all white light to enable acquisition of a background signal that is equivalent to acquiring a background with the light turned off. RS spectra are acquired by placing the optical probe in contact with acetaminophen to calibrate the wavenumber axis. For DRS, a white light spectrum is acquired from an 80% reflectance standard (Spectralon, SRS‐80‐010; Labsphere, North Sutton, NH) after placing an absorptive filter with optical density 0.6 (NE206B, Thorlabs Inc., Newton, NJ) in the light path. The reflectance standard measurement is acquired by placing the optical probe at a fixed height above the surface of the reflectance standard corresponding to maximum reflected light intensity.

### 2.3 Data Acquisition

Data acquisition with this system is controlled by a script written in MATLAB (MathWorks, Natick, MA). The functions provided in a software development kit (SDK) from Andor Technology Ltd. were used to control the Raman spectrograph and the CCD temperature, operate the shutter, and configure Raman data acquisition. An instrument driver was also used to control the DRS spectrometer, set the parameters for spectral acquisition, and read the DRS spectra. The MATLAB scripts for these two drivers were integrated and functions were created to control the entire system in a single script. Each system component—laser, CCD, and switch—is software‐controlled and triggered as needed during acquisition. The detector is cooled to −70°C before spectra are acquired. The total acquisition time depends on the integration time specified for both RS and DRS, with 1 s added in when switching from one light source to another to avoid interference from the previous light source. The laser power measured at the sample from the probe is typically 45% of the power measured from the 785 nm laser. Once all components of the multimodal system are initialized, the probe is placed in contact with the sample. With a single click of a button preset in the code, RS and DRS measurements are acquired and saved sequentially. With the light input as the laser, a Raman spectrum is acquired. Following completion of Raman spectra, the switch automatically changes the input to the halogen lamp to enable DRS spectral acquisition. All spectra are displayed on the computer screen in real‐time upon acquisition.

### 2.4 Data Analysis

#### 2.4.1 Diffuse Reflectance Spectroscopy

The diffuse reflectance spectrum is calculated by dividing the background‐subtracted white light intensity spectrum by the background‐subtracted intensity measurement from the 80% reflectance standard. While not shown here, we utilize a lookup table (LUT)‐based inverse model [37] to fit the DRS spectra and quantify optical properties such as vascular oxygenation, total hemoglobin concentration, and tissue scattering.

#### 2.4.2 Raman Spectroscopy

The wavenumber axis is calibrated according to the Raman spectrum of acetaminophen, and analysis is restricted to the fingerprint region between 600 and 1800 cm^−1^. The Raman spectra are subject to various spectral preprocessing steps, including baseline correction to remove noise, median filtering to remove cosmic spikes, and vector normalization to reduce the impact from laser power variations. Baseline correction is accomplished by fitting a fifth‐order polynomial to the Raman spectrum, which removes the background autofluorescence [21, 38]. Since Raman instruments that use CCD detectors are prone to random spikes induced by cosmic rays, median filtering is used to filter these spikes from the signal. [39] Vector normalization is performed to minimize variations in laser power at the sample [21]. After pretreatment of the Raman spectra, multivariate analysis can be applied to extract relevant biological information as we have demonstrated previously [21, 22, 40].

### 2.5 System Characterization

#### 2.5.1 Human Skin In Vivo Measurements

We used the multimodal system to acquire spectral measurements from the skin on the forearm of a human volunteer. DRS spectra were acquired with an integration time of 0.5 s. To ensure we were within the maximum limits for laser exposure for RS, we computed the maximum permissible laser exposure according to American National Standard Institute (ANSI) Z136.1‐2022 [41]. For a laser power of 40 mW at the sample, and with the radius of our laser beam being 0.05 cm, the calculated maximum laser intensity is 5 W/cm^2^, while the maximum permissible exposure (MPE) intensity at the skin is 0.42 W/cm^2^. We calculated the maximum permissible laser intensity based on these settings, as described earlier [42], and determined that an exposure time of 5 s keeps us well within the permissible limits for laser exposure in this study for a laser power of 40 mW at the skin surface.

#### 2.5.2 Two‐Layer Tissue Phantom Measurements

We also carried out validation measurements on a two‐layer tissue phantom made from chicken fat and muscle. The chicken fat, which was cut to a thickness of 5 mm, was incubated in green dye to detect absorption for DRS measurements. The dye was diluted to 0.1% of the original concentration before use in these experiments. The chicken muscle tissue was cut in varying thicknesses from 0.5 to 5.9 mm (0.5, 1.1, 1.7, 2.3, 2.9, 3.5, 4.1, 4.7, 5.3, and 5.9 mm). We created the phantom by overlaying one piece of muscle tissue on the fat tissue at a time and obtained spectral measurements. We used an integration time of 0.5 s to obtain DRS spectra and 5 s for RS measurements, with a laser power of 40 mW at the surface of the phantom. A total of five DRS spectra and five RS spectra were acquired and averaged for each combination of a muscle layer and fat.

## 3 Results

### 3.1 Power Measurements

**FIGURE 3:**
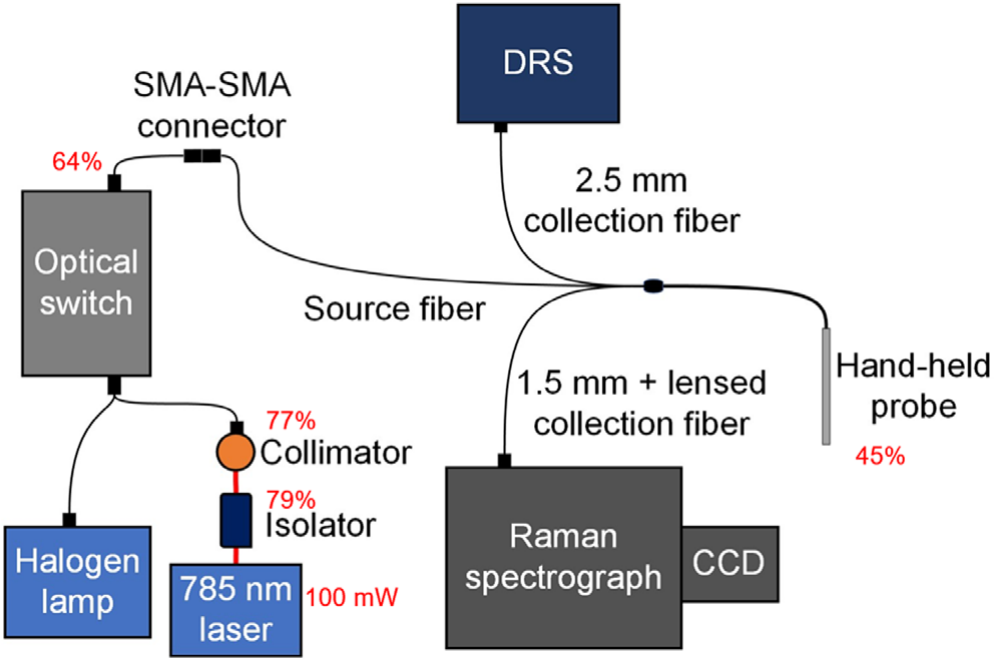
Schematic of the multimodal spectroscopy system showing the parts of the system, and the percentage of power measured relative to the laser output of 100 mW after propagating through each component in the system. Power was measured from the 785 nm laser, isolator, collimator, switch output fiber, and hand‐held probe.

Laser power was measured at different points along the illumination path beginning from the laser module through the optomechanical components and ending at the tissue end of the optical probe (Figure 3). The ACC current was adjusted to deliver a laser power of 100 mW. Power measurements at each critical component as percentages of the laser output power are presented in Figure 3. The power measured at the isolator and collimator was 79% and 77% of the laser power, respectively. After traveling through the optical switch, the power was 64% of the original output. At the tissue end of the hand‐held probe which would be in contact with the sample under investigation, the power measured was 45% of the laser power. The 785 nm laser used for Raman excitation in the system can be operated via push buttons on the laser module or completely remotely via the RS232 port. We integrated commands for control via the latter option into the MATLAB code. The output from the laser had to be aligned with the isolator and collimator so that the signal would not drop significantly before exiting the probe.

### 3.2 Probe Fibers

**FIGURE 4:**
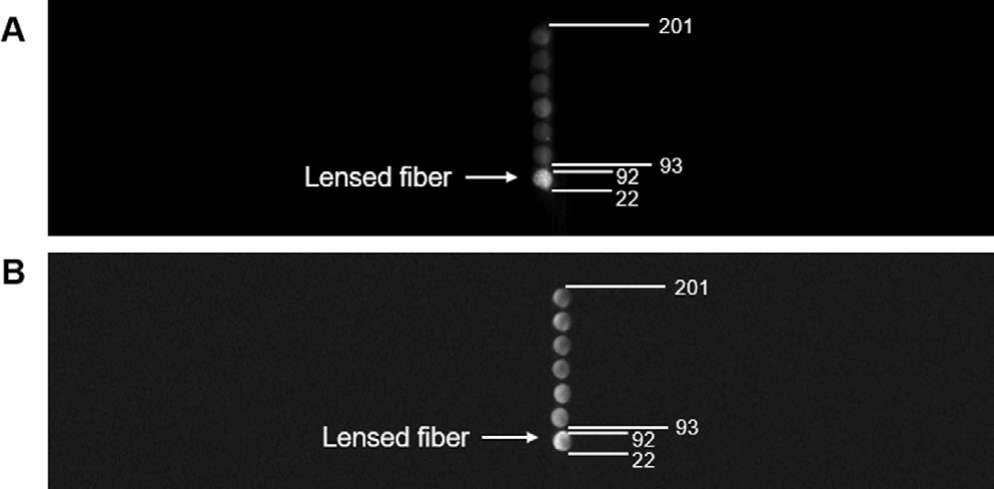
Alignment of probe fibers on the CCD with the lensed fiber labeled for each set of the spatially offset group of fibers. (A) 1.5 mm offset collection fibers imaged using the acetaminophen sample and 785 nm laser. (B) 2.5 mm offset fibers imaged using a reflectance standard and halogen lamp as the light source.

Figure 4 presents images of the collection fibers aligned at the entrance to the Kymera 328i imaging spectrograph collected with a 1024 × 256 pixel CCD. Both source–detector offset configurations contain a lensed collection fiber for superficial measurements along with six other fibers for subsurface measurements. The Raman collection fibers (Figure 4A) consist of six spatially offset fibers at 1.5 mm from the source and one lensed collection fiber (last fiber in the column). The image of the fibers was formed by acquiring Raman scattering from acetaminophen. The lensed fiber appears visibly brighter compared to the spatially offset fibers. In this example, we vertically bin the rows from 22 to 92 to acquire measurements from the zero‐offset lensed fiber, and rows from 93 to 201 to obtain data using the spatially offset fibers. Figure 4B illustrates the DRS collection fibers, which are located at 2.5 mm from the source. Again, the lensed fiber appears brighter than the spatially offset fibers. The DRS fibers are shown here for illustration only. For spectral acquisition, the DRS leg is connected to a USB spectrometer (3648 × 1 array of pixels) with a slit height of 1.2 mm. This corresponds to four central spatially offset fibers and therefore does not include the lensed fiber.

### 3.3 Diffuse Reflectance and Raman Spectra

**FIGURE 5:**
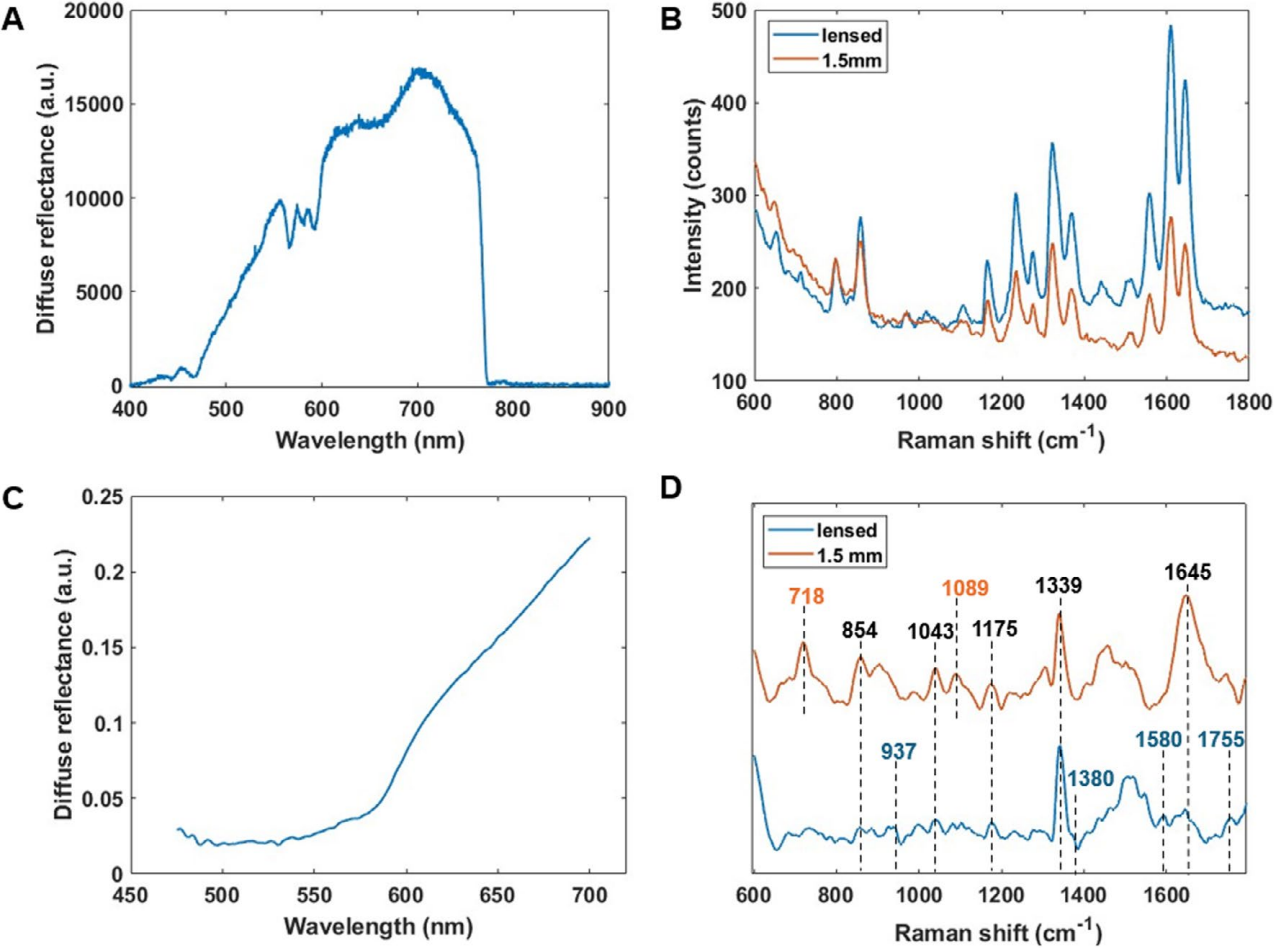
Sample spectra acquired using the multimodal spectroscopy system. (A) White light intensity spectrum recorded from a reflectance standard using the 2.5 mm offset probe fibers. (B) Raman spectra of acetaminophen obtained using the lensed Raman and 1.5 mm SORS probe fibers. (C) Reflectance spectrum of human skin measured in vivo using the 2.5 mm offset probe fibers. (D) Raman spectra of in vivo human skin (vertically offset for clarity) obtained using the zero‐offset ball lens fibers and the 1.5 mm spatially offset probe fibers, with the common peaks labeled in black. Peaks unique to either fiber configuration are labeled using the respective colors.

Figure 5A presents the background‐subtracted reflected light recorded from the 80% reflectance standard. The DRS spectrometer can acquire spectra in the wavelength range of 375–1100 nm. However, due to the presence of a short‐pass filter in the excitation fiber, there is negligible light beyond 800 nm. Figure 5B presents lensed and spatially offset Raman spectra acquired from acetaminophen. These spectra were obtained with an integration time of 0.25 s for DRS and 1 s for RS, with laser power of 40 mW at the sample. Separate regions corresponding to the spatial position (Figure 4A) of the lensed and spatially offset fibers on the CCD are set up within the code to acquire lensed Raman and spatially offset Raman spectra. Given the nature of the sample (solid block), all acetaminophen peaks are visible in both the lensed and spatially offset fibers. We also acquired optical spectra from human skin (Figure 5C,D). Both DRS and RS spectra shown represent an average of five measurements from the same location. The low values of reflectance observed in the DRS spectrum are due to the presence of melanin in the volunteer's forearm. Unlike the measurements on acetaminophen, there are clear differences in the peaks visible in the lensed Raman and SORS spectra, especially in the range of 600–1300 cm^−1^. Peaks at 1043 cm^−1^ (proline from collagen), 1175 cm^−1^ (cytosine, guanine from nucleic acid), 1339 cm^−1^ (CH_2_/CH_3_ wagging and twisting mode in collagen, nucleic acid and tryptophan), 1458 cm^−1^ (CH_2_/CH_3_ deformation of lipids and collagen), and 1645 cm^−1^ (α‐helix of Amide‐I) were present in both Raman spectra. The prominent peaks obtained from the lensed and 1.5 mm spatially offset probe fibers are shown in Table 1. While both sets of fibers show several peaks, the intensity of peaks was lower in the lensed fibers compared with the SORS spectra. Interestingly, we were able to identify melanin‐peaks from the lensed probe at 1380 and 1580 cm^−1^ [44], which were not as apparent in the 1.5 mm fiber spectra.

**TABLE 1:** Peak assignments and corresponding vibrational modes of Raman spectra obtained from the lensed and 1.5 mm fiber optic probe on the human forearm [43].

Wavenumber (cm^−1^)	Tentative biomolecule	Raman vibrational modes	Lensed	1.5 mm
718	Lipid/nucleic acid	C–N (membrane phospholipid head)/nucleotide		X
733	Lipid	Phosphatidylserine	X	
854	Protein	Ring breathing tyrosine (proteins)	X	X
903	Nucleic acid	Phosphodiester, deoxyribose		X
937	Protein	C–C backbone (collagen assignment)	X	
980	Protein/lipid	C–C stretching b‐sheet (proteins) = CH bending (lipids)		X
1002	Protein	C–C aromatic ring stretching of Phenyl alanine	X	
1043	Protein	Proline (collagen assignment)	X	X
1089	Nucleic acid	Symmetric PO_2_^−^ stretching vibration of the DNA		X
1175	Nucleic acid	Cytosine, guanine	X	X
1221	Protein	Amide III (b‐sheet)		X
1305	Lipid, nucleic acid	CH_2_ deformation (lipid), adenine, cytosine		X
1339	Protein/nucleic acid	CH_2_/CH_3_ wagging & twisting mode in collagen, nucleic acid & tryptophan	X	X
1380	Melanin	Linear stretching of the C–C bonds within the rings [44]	X	
1404	Protein	n(C=O)O– (aspartic & glutamicacid)		X
1458	Lipid/protein	CH_2_/CH_3_ deformation of lipids & collagen	X	X
1510	Nucleic acid	A (ring breathing modes in the DNA bases)	X	X
1580	Melanin	In‐plane stretching of the aromatic rings [44]	X	
1645	Protein	Amide I (a‐helix)	X	X
1736	Lipid	C=O ester (lipids)		X
1755	Lipid	C=O stretching	X	

We determined the signal‐to‐noise ratio (SNR) for the spectra acquired with the spatially offset fibers and the zero‐offset ball lens fiber. The experimental SNR for a Raman peak is calculated by dividing the average peak height by the standard deviation of that peak across multiple measurements [45]. We chose the peak at 1339 cm^−1^ for our SNR calculation, as it was a common visible peak for both fiber types. For the lensed fiber, we calculated the SNR to be 10, which was slightly higher than 5.8 for the 1.5 mm spatially offset fibers.

### 3.4 Probe Sampling Depth

**FIGURE 6:**
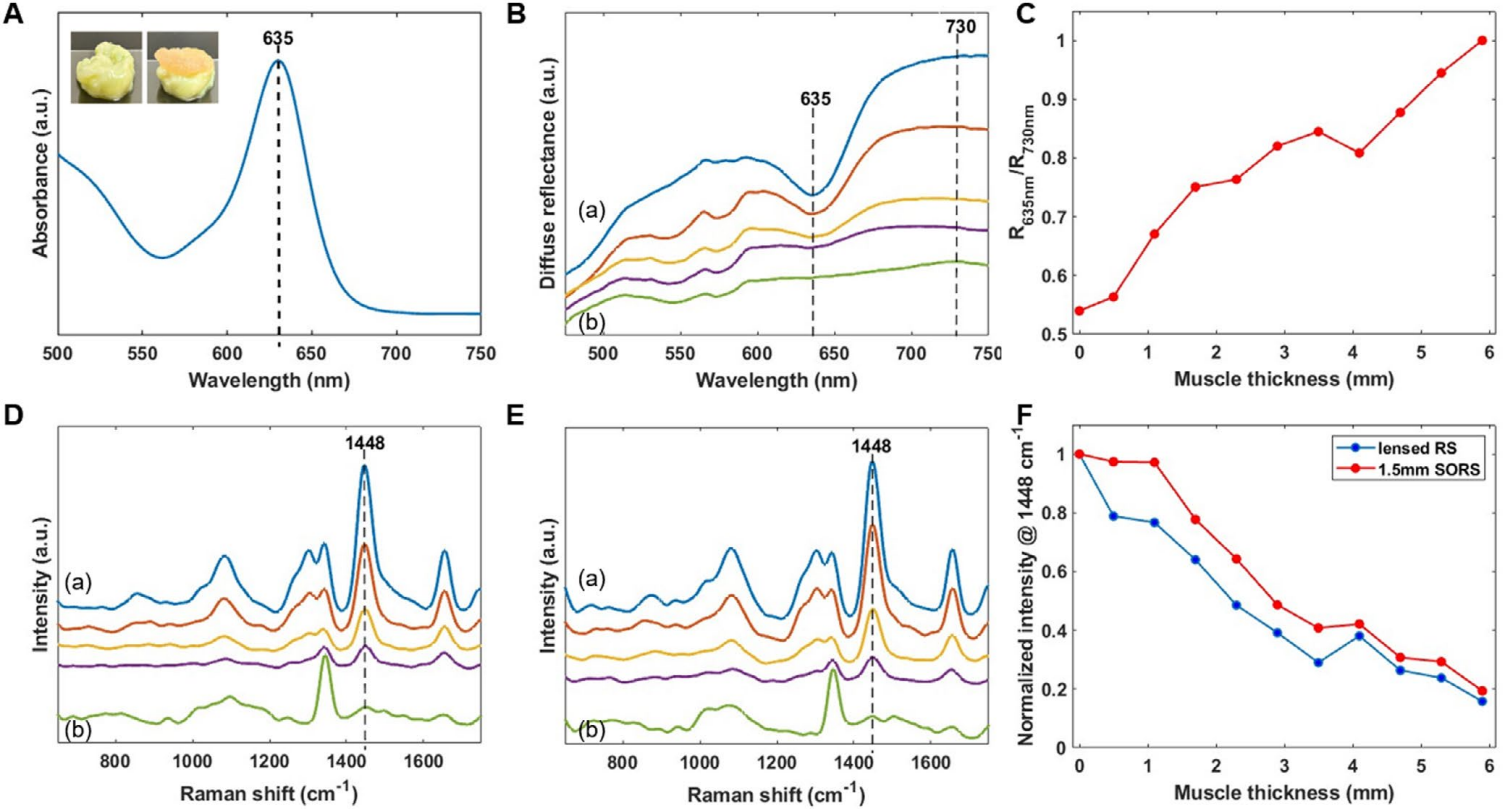
(A) Absorption profile of green dye, with an inset of the two‐layer tissue phantom, made from chicken fat and muscle tissue, used to detect depth‐dependent changes using DRS and RS. (B) Diffuse reflectance spectra acquired from the tissue phantom showing changes in the dip in the reflectance spectrum corresponding to green dye absorption—(a) represents fat only while (b) represents the thickest layer of muscle tissue only (5.9 mm) and the spectra in between are increasing thicknesses of muscle tissue on fat tissue. Spectra have been vertically offset for visualization purposes. (C) Ratio of reflectance intensities of green dye at an absorbing wavelength to a nonabsorbing wavelength as a function of muscle tissue layer thickness. (D) Zero‐offset lensed Raman spectra and (E) 1.5 mm spatially offset Raman spectra obtained from the two‐layer tissue phantom: (a) represents fat only while (b) represents the thickest layer of muscle tissue only (5.9 mm) and the spectra in between are increasing thicknesses of muscle tissue on fat tissue. Spectra have been vertically offset for visualization purposes. (F) Normalized Raman peak intensities at 1448 cm^−1^ as a function of muscle tissue layer thickness for lensed RS and SORS.

Since changes in tissue morphology or biochemical constituents may be depth dependent, we wanted to see how the detection of certain components changes with depth using DRS and RS using a two‐layer phantom made from chicken fat and muscle tissue. The chicken fat was soaked in green food color for a period of 30 min. The absorption profile of the green dye used in this experiment, with an absorption peak at 635 nm, as well as an inset of the dyed chicken fat and the two‐layer phantom is shown in Figure 6A. Figure 6B presents the DRS spectra acquired from chicken fat alone (blue line (a)) and for increasing thickness of muscle overlaid on fat. Increasing the muscle thickness eventually leads to the disappearance of the absorption trough in the DRS spectrum (Figure 6C). We calculated the ratio of the wavelength at which this trough occurs (635 nm) to a nonabsorbing wavelength (730 nm) to quantify the disappearance of the signal. The reduced contribution from green dye with increasing muscle thickness results in lower absorption at 635 nm and therefore an increase in the ratio of *R*_635_/*R*_730_. Even at a depth of 2.3 mm of muscle over fat, we were still sensitive to reflectance from the green dye in the fat layer. We also plotted Raman spectral data obtained with the zero‐offset lensed collection fibers (Figure 6D) as well as the 1.5 mm spatially offset collection fibers (Figure 6E). Chicken fat and muscle tissue display characteristic Raman features [46, 47], which we detected using our system. When the probe was placed in contact with only fat tissue (spectrum labelled (a)), the intensity of the Raman peak at 1448 cm^−1^ (CH_2_ bending of lipids) is high. The intensity decreases remarkably when measurements are taken with even the thinnest layer of muscle tissue (0.5 mm) placed on the fat tissue (Figure 6F). This is more apparent for the zero‐offset lensed fibers compared to the spatially offset fibers. For muscle thickness greater than 2 mm, we observe that the spatially offset fibers retain ~64% of the original intensity, while the zero‐offset fibers are only able to detect ~49% of the original intensity.

## 4 Discussion

Several studies have demonstrated that combining multiple spectroscopic modalities results in greater accuracy in disease diagnosis [48]. Zângaro et al. [49] designed and built an instrument that combined laser‐induced fluorescence spectroscopy (LIFS) corresponding to 11 different excitation wavelengths and DRS, and was later deployed for diagnostic studies of the esophagus [50]. Improved versions of this instrument featuring shorter acquisition times [51, 52] were later used in studies of the oral cavity [53] and to diagnose breast cancer [54]. Tunnell and colleagues developed a modified version of this instrument that combined DRS and LIFS excitation at only two wavelengths for detection of melanoma and nonmelanoma skin cancers [55]. These systems utilized the same set of collection fibers and hence the same source–detector separation for both DRS and LIFS because both sets of spectra were acquired over a similar wavelength range. Šćepanović et al. [56] developed a unified optical probe that could acquire DRS, RS, and LIFS spectra to detect morphological features of vulnerable atherosclerotic plaques. This instrument used different sets of collection fibers for DRS and RS; however, the same source–detector separation was used for all three modalities even though RS was acquired at much longer excitation wavelengths. Here, we demonstrate a combined DRS and RS instrument that utilizes separate source–detector offsets for each modality that recognizes the different sampling depths enabled by the excitation wavelengths of each modality. Our goal was to develop an optical probe and multimodal system with similar zones of acquisition for both DRS and RS.

The SNR plays a key role in any spectroscopic system's ability to provide meaningful data, especially in RS where the signal is naturally weak. A high SNR means the signal is much clearer than the background noise, making it easier to detect and analyze. Fitzgerald et al. [57] calculated the SNR of their Raman system at 1440 cm^−1^, as this was the most prominent peak observed in their biological sample, milk. In our study, we computed the SNR for our Raman system using measurements taken from the forearm of a volunteer. While our SNR values seemed relatively low, Raman scattering is an inherently weak process, especially in biological samples.

The sampling depths for both DRS and RS in the multimodal probe depend on the separation between the illumination and collection zones as well as the wavelengths being used. A shorter offset is used for RS (1.5 mm) than for DRS (2.5 mm) to enable depth matching between the two modalities, since the RS excitation is in the near infrared wavelength range, while DRS spectra are acquired in the visible range. Studies performed on a two‐layer tissue phantom using chicken fat and muscle show reasonably good sensing with both spatially offset RS and DRS at up to a depth of 4 mm. However, there are drawbacks with the use of chicken breast in such experiments. Chicken tissue is not very representative for human tissue since scattering and absorption in chicken tissue are much lower [58]. This could affect sampling depth estimations, causing us to detect signal at greater depths with the two‐layer phantom than we would in human tissue. Nevertheless, these phantom measurements allow an understanding of differences in sampling between the zero‐offset lensed fiber and the spatially offset fibers in RS. Our measurements on the skin of a human volunteer also demonstrate that biomolecular peaks related to melanin show up prominently in the lensed fiber spectra but are much weaker in the spatially offset fibers. The presence of an integrated zero‐offset fiber within our spatially offset probe also allows us to use these measurements as a reference to ensure that data are being acquired from the surface of the sample. The intensity of the measurements acquired using the lensed fiber was the highest when the probe was in contact with the sample and decreased once there was distance between the probe contact end and the sample surface.

The combination of DRS and RS could potentially provide a powerful tool to measure treatment response in vivo. Given the vital role that oxygen plays in response to different types of therapy, DRS is an obvious candidate to measure treatment response. However, measures of tumor oxygenation alone can be insufficient because reoxygenation is observed in both responsive and resistant tumors. A complementary modality, such as RS, that measures other aspects of the tumor microenvironment known to change in response to therapy, such as modifications of the extracellular matrix [59], immune cell recruitment [22], and changes to lipid saturation [60] could help differentiate treatment responders from nonresponders better than measures of tumor oxygenation alone.

## 5 Conclusions

We have developed an integrated multimodal spectroscopy system for simultaneously collecting diffuse reflectance and Raman spectra using spatially offset fibers for both modalities. Using an optical switch to alternate between light sources, we were able to rapidly acquire spectra from each modality. Proof‐of‐concept in vivo measurements from healthy human skin demonstrate that there are clear differences in the Raman peaks obtained from deeper‐sensing spatially offset Raman fibers and zero‐offset lensed Raman fibers. We were able to identify Raman signals related to proteins and lipids at 937 and 1755 cm^−1^ for the conventional backscattering configuration, and lipids and nucleic acids at 718 and 1089 cm^−1^ for the spatially offset setting. Combining DRS and RS into a single instrument could provide a powerful method for rapid and noninvasive evaluation of treatment‐induced changes in the tumor. Both modalities used here are label‐free and do not require the use of exogenous contrast agents. Repeated measurements can be acquired over several days or weeks, without adversely affecting tissue.

Future plans for this multimodal spectroscopy system include assembling the entire system on a portable cart and moving it to a clinic, where it can be used to monitor real‐time tumor response to therapies, such as chemo, radiation, and immunotherapy. For accessible tumors in the oral cavity and on the skin, the probe can be used with its current design; however, for less accessible tumors like those in the larynx, the probe will have to be redesigned to be able to be compatible with endoscopes used in clinical practice. We also plan to design and develop a probe with an inverse spatially offset configuration that can be integrated into this system and enable the use of lower power at the sample due to distributed illumination, which would be suitable for in vivo applications.

## Conflicts of Interest

The authors declare no conflicts of interest.

## Data Availability

The data that support the findings of this study are available from the corresponding author upon reasonable request.
